# The effectiveness of rigid pericardial endoscopy for minimally invasive minor surgeries: cell transplantation, epicardial pacemaker lead implantation, and epicardial ablation

**DOI:** 10.1186/1749-8090-7-117

**Published:** 2012-11-09

**Authors:** Takehiro Kimura, Shunichiro Miyoshi, Kazuma Okamoto, Kotaro Fukumoto, Kojiro Tanimoto, Kyoko Soejima, Seiji Takatsuki, Keiichi Fukuda

**Affiliations:** 1 Department of Cardiology, Keio University School of Medicine, Tokyo, Japan; 2 Department of Cardiovascular surgery, Keio University School of Medicine, Tokyo, Japan; 3 Department of Cardiology, Kyorin University Hospital, Tokyo, Japan

**Keywords:** Rigid pericardial endoscopy, Minimally invasive surgery, Cell transplantation, Epicardial pacemaker lead implantation, Epicardial ablation

## Abstract

**Background:**

The efficacy and safety of rigid pericardial endoscopy as the promising minimally invasive approach to the pericardial space was evaluated. Techniques for cell transplantation, epicardial pacemaker lead implantation, and epicardial ablation were developed.

**Methods:**

Two swine and 5 canines were studied to evaluate the safety and efficacy of rigid pericardial endoscopy. After a double pericardiocentesis, a transurethral rigid endoscope was inserted into the pericardial space. The technique to obtain a clear visual field was examined, and acute complications such as hemodynamic changes and the effects on intra-pericardial pressure were evaluated. Using custom-made needles, pacemaker leads, and forceps, the applications for cell transplantation, epicardial pacemaker lead implantation, and epicardial ablation were also evaluated.

**Results:**

The use of air, the detention of a stiff guide wire in the pericardial space, and the stretching of the pericardium with the rigid endoscope were all useful to obtain a clear visual field. A side-lying position also aided observation of the posterior side of the heart. As a cell transplantation methodology, we developed an ultrasonography-guided needle, which allows for the safe visualization of transplantation without major complications. Pacemaker leads were safely and properly implanted, which provides a better outcome for cardiac resynchronizing therapy. Furthermore, the success of clear visualization of the pulmonary veins enabled us to perform epicardial ablation.

**Conclusions:**

Rigid pericardial endoscopy holds promise as a safe method for minimally invasive cell transplantation, epicardial pacemaker lead implantation, and epicardial ablation by allowing clear visualization of the pericardial space.

## Background

Although tissue engineering research has developed various techniques to establish stem cell differentiation, there have been no established techniques to transplant these resources. The intracoronary infusion of cell resources has been clinical used for acute myocardial infarction [1], ischemic heart disease [2], and nonischemic cardiomyopathy [3]. However, this approach faced major complications due to microvascular embolization [4]. Intramyocardial delivery, which is a direct injection into the myocardium, is more beneficial in cell retention and therapeutic effects compared to systemic, intracoronary, or intravenous infusion methods [4,5]. It can be achieved by a transepicardial or transendocardial approach [6]. Transepicardial delivery is usually performed by way of a sternotomy during coronary artery bypass [7], subdiaphragmatic access [8], or coronary sinus [9]. Transendocardial delivery requires a retrograde left ventricular approach via the femoral artery [10]. For this purpose, the NOGA XP Cardiac Navigation system™ (Biologics Delivery Systems Group, Cordis Corporation, Diamond Bar, CA, USA) [11] and the MyoStar™ catheter (BiosenseWebster, Diamond Bar, CA, USA) [12] are used as cell delivery navigation systems. These systems allow recognition of the specific areas upon which to deliver laser energy [13], genes [14], and cells [15] under magnetic and electromechanical guidance; moreover, these methods have been reported to be effective for ischemic [16] and nonischemic disease [17]. However, there is still a risk of microvascular embolization [18], and the limitations due to positional errors during mapping remain to be solved.

Therefore, we have evaluated the feasibility of flexible pericardial endoscopy as a minimally invasive intramyocardial delivery method with direct visualization of the pericardial space [19]. The flexible endoscope was inserted from the subxyphoid area by using the Seldinger technique under fluoroscopic guidance. The small pericardial space was inflated by air to obtain a clear visual field to maintain a certain distance between the endoscope and the heart. The flexible pericardial endoscope led to no acute or chronic complications. Too much air in the pericardial space induced transient blood suppression, which was called “air tamponade”. This was immediately resolved by suctioning the excess air. The endoscopic image was so vivid no other guidance systems were necessary for minor surgery applications.

Direct endoscopic imaging is free from the limitations of mapping error and can be a promising technique for minor surgery in the pericardial space. To explore these advantages and to establish pericardial endoscopic surgery, we evaluated the use of rigid pericardial endoscopy for a better visual field and easier access to the pericardial space. In this study, we invented a safer ultrasonography-guided cell transplantation system. We focused on further optimization of epicardial approach devices to treat arrhythmias, which included epicardial pacemaker lead implantation and epicardial ablation.

## Methods

All of the experimental protocols were approved by the institutional ethical committee.

### A double sheath puncture procedure

Five canines weighing 18–25 kg and 2 swine weighing 30–35 kg were studied. Following the infusion of 0.5 mg/kg pentobarbital, the animals were intubated and ventilated with room air by a constant-volume cycled respirator (Model SN-480-3, Shinano Inc., Tokyo, Japan). The animals were anesthetized with 1.5% halothane, and a 50 ml/h normal saline infusion was used to compensate for body fluid loss and drug delivery. The right femoral arterial blood pressure, electrocardiogram, and pulse oximetry were continuously monitored during the procedures.

After local anesthesia, sheaths were placed in the pericardial space by using the modified Seldinger technique [19]. An 18 Gauge needle for epidural anesthesia was used for percutaneous pericardiocentesis from the epigastric fossa under ultrasonography and fluoroscopy guidance. After safely puncturing the pericardium with a puff of contrast medium, a guide wire (outer diameter (OD) = 0.35 mm) was inserted into the pericardial space. The cutaneous hole was gradually upsized by several polypropylene dilators (8 French, 12 French). Afterwards, 2 types of sheaths, which are described below, were inserted. For a double sheath maneuver, the second sheath was inserted by repeating the same procedure. The second puncture site was directed as far as possible from the first site to avoid interference of both sheaths.

### Device design

**Figure 1:**
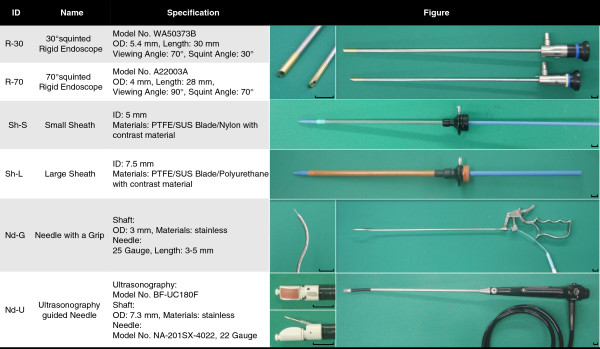
**Characteristics of the devices for pericardial endoscopic surgery.** We developed unique tools for pericardial endoscopic maneuvers. The ID, name, specification, and figure of devices are shown. R-30 and R-70 were originally designed for transurethral cancer resection with a 30 and 70° squinted visual field. Sh-L and Sh-S are 15 French and 22.5 French sized sheaths, respectively, with guide wires clamps. Nd-G is the cell transplantation needle with a grip and Nd-U is visible under ultrasonography. Black scale: 1 cm; ID: inner diameter; OD: outer diameter; PTFE: polytetrafluoroethylene.

We used several types of rigid endoscopes, sheaths, needles, and forceps, as shown in Figure 1. These instruments were designed as prototypes for rigid pericardial endoscopic surgery.

Two kinds of rigid endoscopes (Model WA50373B and A22003A, Olympus Medical Systems Corporation, Tokyo, Japan) were used (Figure 1, R-30, R-70). These endoscopes were originally designed for transurethral resection of cancers. These endoscopes provided a 30° (R-30) and a 70° (R-70) cross-eyed visual field. The R-30 endoscope provided a 70° viewing angle and the R-70 endoscope provided a 90° viewing angle. The viewing angles were sufficiently wide to observe the surface of the heart at close range. Other information, such as model numbers, sizes, and specifications are shown in Figure 1.

We used 2 types of handmade sheaths: a smaller sheath (Figure 1, Sh-S) for the rigid endoscopes and a larger sheath (Figure 1, Sh-L) for forceps and needles. The sheaths were fluoroscopically visible and were equipped with a check valve to seal the air in the pericardium. A clasp for the guide wire was also attached to prevent the unintentional extraction of the guide wires.

We arranged 2 types of guide wires; a soft, plastic, readymade guide wire was used for the Seldinger technique, which was later replaced by a stiff guide wire (Radifocus™ Guide Wire M, Terumo Corporation, Tokyo, Japan) that was detained inside of the pericardial space and fixed by the wire clasp during sessions.

Two types of needles for cell transplantation were designed by modifying abdominal forceps. A needle with a grip (Figure 1, Nd-G) was inserted through Sh-L; this consisted of a 25 Gauge needle attached to the inside of the metal forceps and connected to a syringe. By grasping the grip, the needle extended 3–5 mm from the tip of the forceps. Moreover, we developed an ultrasonography-guided needle (Figure 1, Nd-U). The tip of the readymade ultrasonographic needle (Model BF-UC180F, Olympus Medical Systems Corporation, Tokyo, Japan) was attached to the hard shaft, and a 23 Gauge needle extended out from the probe. The probe could be curved 90° in either direction to position the probe on the surface of the heart.

### Manipulation establishment and safety evaluation

After a double sheath insertion, the rigid endoscopes were advanced through the Sh-S sheath, and forceps or needles were inserted through Sh-L. The pericardial space was inflated with air, as previously reported [19]. Optimization of the devices and maneuvers for a clearer visualization of the pericardial space were performed. Furthermore, a cell transplant demonstration, epicardial pacemaker lead implantation, epicardial ablation, and a safety evaluation of a continuous carbon monoxide supply device were evaluated as described below. Complications such as hemorrhages, arrhythmia, and hemodynamic data were continuously monitored throughout the procedures.

### Cell transplant demonstration

To simulate the clinical implications for cell transplantation, 24 μm silicon beads (Catalog No.35-5B, Thermo Scientific, Kanagawa, Japan) were transplanted by Nd-G and Nd-U in 2 canines. The silicon beads were mixed with indocyanine green, a contrast medium for fluoroscopy (Iomeron™, Eizai Co.,Ltd., Tokyo, Japan), and a contrast medium for ultrasonography (Levovist™, Bayer-Schering, Berlin, Germany) to identify the infiltration in the myocardium. A total of 5 ml of the beads medium (10^5^-10^6^ beads/mL) was transplanted by puncturing the myocardium 20 times. After the procedure, the animals were sacrificed and the hearts were evaluated macroscopically and microscopically with a fluorescence filter (Model PB0007, Asahi Spectra Co., Ltd. Tokyo, Japan).

### Epicardial treatment for arrhythmia

In cardiac resynchronizing therapy, an epicardial approach with open chest surgery can be considered in cases where appropriate positioning of pacing leads via the coronary sinus is difficult. However, as a minimally invasive alternative, various pacemaker leads were implanted by rigid pericardial endoscopy. The intraventricular pacing lead (Model 5076, Medtronic, Minneapolis, MN, USA), the epicardial temporary pacing lead (Model 6491, Medtronic, Minneapolis, MN, USA) and the epicardial, sutureless, myocardial lead (Model 5071–35, Medtronic, Minneapolis, MN, USA) were used. The intraventricular pacing lead was implanted with a deflectable stylet (Model 6094–58, Medtronic, Minneapolis, MN, USA) and fixed in the left ventricular free wall of the canines. The epicardial temporary pacing lead was transplanted in the right ventricle with a needle holder. Implantation of the epicardial, sutureless, myocardial lead was attempted by screwing the lead into the myocardium through a 14 mm sized sheath in a swine. After implantation, the lead was connected to the stimulator (Model 5348, Medtronic, Minneapolis, MN, USA) and parameters of sensitivity and pacing threshold were measured.

Furthermore, applications for epicardial ablation were evaluated. Epicardial radiofrequency catheter ablation is currently the established, intracardiac approach for incurable arrhythmia [20]. Attempts to obtain a clear visualization of the heart to perform ablation were evaluated in the canines. Complications caused by clamping the pulmonary veins were monitored.

### Intrapericardial pressure monitoring

A 60 kg swine was used to examine the relationship between intrapericardial pressure and hemodymanics. After a single pericardiocentesis, the continuous carbon dioxide infusion device (Model UHI-3; Olympus Medical Systems Corporation, Tokyo, Japan) used for pneumoperitoneum during laparoscopic surgery was connected to 8 French sheaths. The intrapericardial pressure, vital signs, fluoroscopy, and the amount of air during the procedure were monitored.

## Results

### Manipulation of pericardial endoscopic surgery

The manipulation of rigid pericardial endoscopes was intuitive even for cardiologists; however, stretching the pericardium with endoscopes and managing body position were important to clearly visualize the narrow pericardial space.

First, a rigid endoscope was inserted into Sh-S from the epicardial fossa to obtain the antero-posterior caudal view. This enabled us to look up the anterior base of the heart from the apical position. Stretching the pericardium by moving the endoscope like a lever allowed us to enlarge the space between the pericardium and the heart so that a better overview of the wide area of the heart could be obtained. By rotating the shaft of the endoscope, both sides of the lateral wall of the heart were observed. The base of the ventricles, atrial appendages and atriums were confirmed by carefully advancing the endoscopes. To observe the posterior and lateral sides of the heart for the pulmonary veins and the coronary sinuses, the body should be positioned in a recumbent position toward the opposite side of the desired target; for instance, a right lateral recumbent position is required for the left pulmonary veins. The shaft was then rotated and gently moved around the heart along the pericardium.

For a clearer visualization of the narrow pericardial space, air injection was the critical technique required, as described in the previous paper [19]. The “air tamponade” gave us enough space for handling the endoscopes. However, detention of the guide wire in the pericardial space was also valuable in preserving the work space. The stiff guide wires served as struts in the pericardium, similar to pitching a tent. This procedure could reduce the amount of air inside of the pericardial space, which resulted in less risk of hemodynamic deterioration.

The images obtained from rigid pericardial endoscopes were vivid enough to identify not only anatomical structures but also small branches of coronary vessels and details of the heart tissue condition. Therefore, minor surgery was able to be performed as follows.

### Cell transplantation

There are no accepted minimally invasive techniques to transplant cells into the myocardium for tissue engineering therapy; however, we have now established secure and precise transplantation tools.

**Figure 2:**
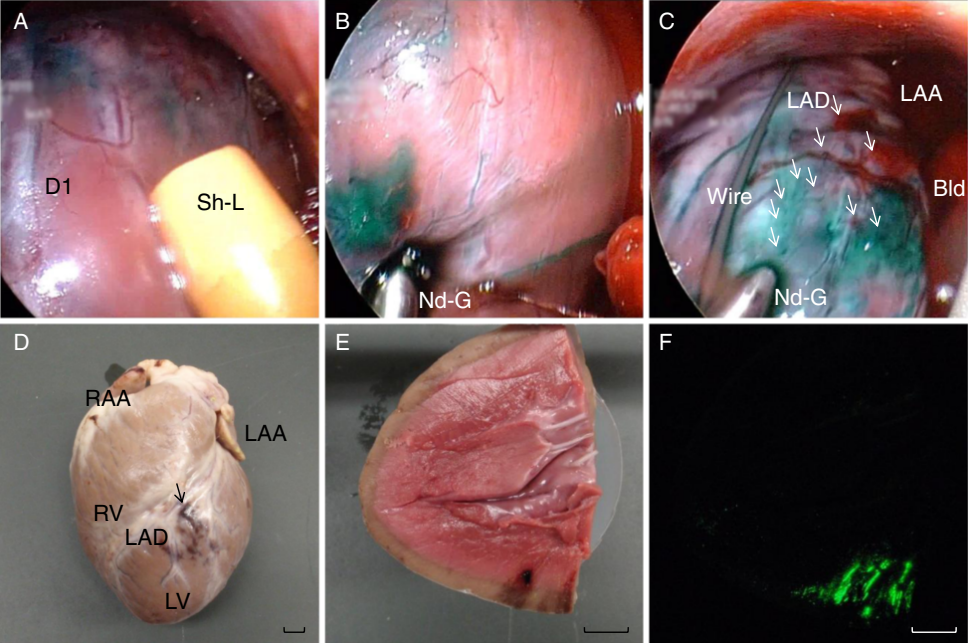
**Demonstration of cell transplantation with needle with grip (Nd-G).** A mixture of silicon beads, indocyanide green, ultrasonographic and fluoroscopic contrast medium was injected into the myocardium by Nd-G. **A**: Two sheaths were inserted into the pericardial space, Sh-S with R-70 and Sh-L with Nd-G. Coronary vessels such as the first diagonal branch (D1) were clearly visualized to avoid injuries. **B**: Nd-G was inserted through Sh-L and placed at the puncture site while avoiding coronary vessels. **C**: Injections were performed 20 times (white arrows). Intraventricular injections due to strong motion artifacts caused a slight hemorrhage (Bld) that spontaneously stopped without hemodynamic change. Macroscopic evaluation (**D**), a cross section of injected sites (**E**), and a cross section with fluorescence filter (**F**) showed that the transmural injection was successfully performed in the anterolateral wall of the left ventricle. Bld: blood; LAA: left atrial appendage; LAD: left anterior descending artery; LV: left ventricle; RAA: right atrial appendage; RV: right ventricle; Wire: a guide wire; Sh-L, Sh-S, Nd-G; as described in Figure 1.

**Figure 3:**
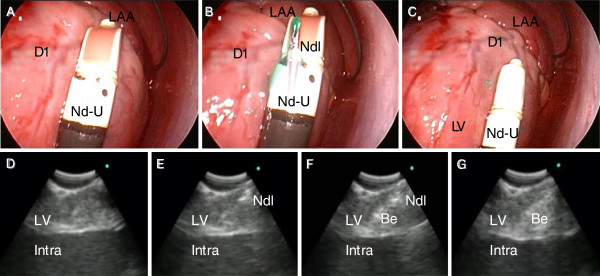
**Ultrasonography-guided cell transplantation.** To avoid intracardial injections, we developed ultrasonography-guided needle forceps (Nd-U). **A**: After a double sheath procedure, R-70 and Nd-U were inserted. **B**: The 23 Gauge needle was extended under ultrasonographic guidance. **C**: Avoiding any coronary vessels, Nd-U was placed on the lateral wall of the left ventricle. **D**: An ultrasonographic image was useful in determining anatomical orientations and major vessels at injection sites. **E**: The needle was extended from the upper right corner of this image. **F**: Beads were securely injected in the myocardium by confirming the location of the tip of the needle. **G**: After injections, bead retention in the myocardium was confirmed by a residue of ultrasonographic contrast medium. Be: a mixture of silicon beads and contrast medium; D1: first diagonal branch; Intra: intra ventricle; LAA: left atrial appendage; LV: left ventricle; Ndl: needle; Nd-U as described in Figure 1.

The double sheath approach enabled us to deliver forceps and endoscopes to desired sites. A mixture of silicon beads, indocyanine green, fluoroscopic contrast, and ultrasonographic contrast medium was transplanted to demonstrate cell transplantation. After insertion of R-70 into Sh-S, Sh-L was inserted into the pericardium next to Sh-S (Figure 2A). Nd-G operating through Sh-L was delivered to a desired puncture site while avoiding small coronary vessels (Figure 2B). The slight compression of the heart by Nd-G did not cause a blood pressure drop or ventricular arrhythmia; however, this helped to stabilize the needle on the heart. By grasping the grip of Nd-G, the needle protruded from the tip, and beads were injected into the myocardium. The injection sites were macroscopically confirmed by indocyanine green and fluoroscopically by the contrast medium. The transplantation was performed 20 times (Figure 2C). Nonsustained ventricular tachycardia was observed during injection without hemodynamic changes. The heart was dissected after the session (Figure 2D), and successful transmural transplantation was confirmed with slight hemorrhage in the myocardium (Figure 2E, F). Minor bleeding was observed when needles penetrated the myocardium, which spontaneously stopped within a minute without any hemodynamic change. Due to a strong cardiac motion artifact, the risk of transmural injection by an accidental intraventricular injection was inevitable by using Nd-G. Therefore, we developed an ultrasonography-guided transplant needle (Nd-U) to secure the intramural injection. The target site of injection was viewed by the rigid endoscope to avoid injury to the coronary arteries (Figure 3A, B). The probe of the ultrasonography was then firmly attached to the surface of the heart (Figure 3C). Ultrasonography gave sufficient information about the myocardium: myocardial thickness, blood vessels, valve positions, and other anatomical characteristics. Once the puncture site was securely confirmed by ultrasonography (Figure 3D), the needle was introduced 3–5 mm from the probe (Figure 3E). The needle was locked and prevented from protruding more than 5 mm as a safety measure. After confirmation of the needle position, the beads were slowly injected (Figure 3F, G). The injected material appeared to be a residue of the ultrasonographic contrast medium. There was no risk of intraventricular injection and blood vessel injury. This procedure enabled us to safely transplant cells intramurally, even safer than open chest surgery.

### Pacemaker lead implantation

Epicardial pacemaker leads are used for cardiac resynchronizing therapy when the anatomy of the coronary sinus is not suitable for the maximum pacing benefit, which currently requires open chest surgery. Therefore, we developed a methodology for minimally invasive endoscopic epicardial pacemaker lead implantation.

**Figure 4:**
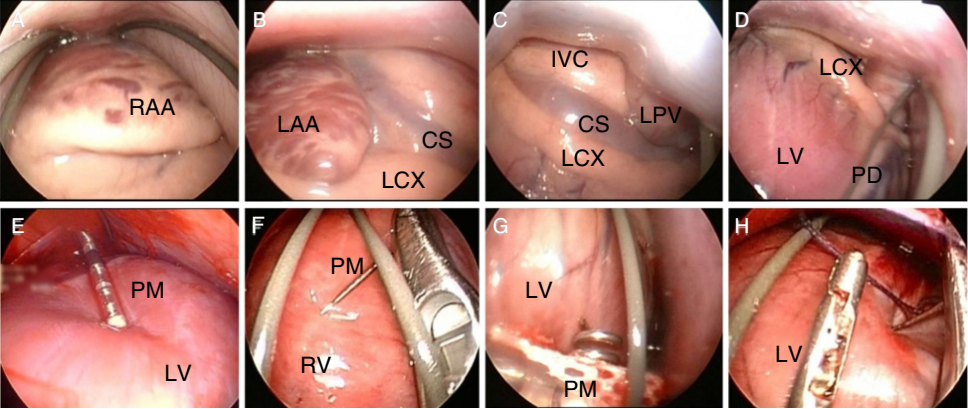
**Epicardial pacemaker lead implantation.** To identify the best position of left ventricular pacing sites, establishing anatomical orientations was critical. **A**: In the dorsal position, the right atrial appendage was confirmed by simply advancing the rigid endoscope toward the top of the pericardium. **B**: In the right lateral recumbent position, the left atrial appendage (LAA), distal of the coronary sinus (CS) and left circumflex artery (LCX) were clearly confirmed. **C**: By moving the rigid endoscope around the heart, the base of the postero-lateral wall of the left ventricle, the inferior vena cava (IVC) and the left pulmonary vein (LPV) were also identified. **D**: By pulling the rigid endoscopes toward the apex, a broad overview of the postero-lateral wall of the left ventricle, which was the appropriate epicardial pacing target in cardiac resynchronizing therapy, was obtained. **E**: The intraventricular pacing lead with a deflectable stylet was securely implanted in the postero-lateral wall of the left ventricle. **F**: The epicardial temporary pacing lead was implanted in the right ventricle (RV). **G**: An attempt was made to implant the epicardial sutureless pacing lead; however, the device was too large (14 mm) and required modifications. **H**: A suture attempt was successfully performed to fix the epicardial pacing leads. CS: coronary sinus; LAA: left atrial appendage; LCX: left circumflex artery; LPV: left pulmonary vein; LV: left ventricle; PD: posterior descending artery; RAA: right atrial appendage; RV: right ventricle.

For successful outcomes of left ventricular pacing, the orientation of the lateral and posterior sides of the heart is important. When the rigid endoscopes were advanced to the top of the pericardium in a dorsal position, the right atrial appendage was observed (Figure 4A). The left atrial appendage, the left circumflex coronary artery, and the distal of the coronary sinus were able to be visualized with the subject in a right lateral recumbent position (Figure 4B). A clockwise rotation of the rigid endoscope enabled us to observe the base of the postero-lateral wall of the left ventricle, the inferior vena cava, and the left pulmonary vein (Figure 4C). By pulling the rigid endoscope toward the apex, the wide area of postero-lateral wall of the left ventricle was observed (Figure 4D). By properly managing appropriate body positions, the whole heart could be observed and coronary sinus-independent pacing sites could be selected without requiring open chest surgery.

To demonstrate pericardial lead implantation, the intraventricular pacemaker lead was securely implanted at the base of the lateral wall of the left ventricle (Figure 4E). The endoscope was held to observe the basal free wall of the left ventricle. The lead was delivered by the deflectable stylet and placed at the base of the left ventricle under fluoroscopic guidance. The lead was screwed into the myocardium without fatal arrhythmia, hemodynamic change, or hemorrhage. Pacing from the implanted lead was successfully performed, and there were no signs of dislodging or pacing failures 30 min after the implantation. Furthermore, the epicardial temporary pacing lead was successfully implanted in the right ventricle (Figure 4F). The lead was delivered by a needle holder through Sh-L, and pacing worked properly (threshold 0.1 V/0.4 msec, sense >20 mV). The use of an epicardial sutureless myocardial pacing lead was also evaluated (Figure 4G). Although the implantation was successful, the maneuver was difficult, and the pacing condition was not sufficient (threshold: 5 V/0.4 msec, sense: 4 mV). Because the device itself was designed for open chest surgery, the outer diameter of the lead handle was so large (14 mm) that it was not suitable for this approach. Further modification of the lead designed for a rigid pericardial endoscope should be considered.

For implantation of epicardial pacing leads (Model 4968, Medtronic, Minneapolis, MN, USA), a suture attempt was successfully performed using round needles (3–0 Vicryl) and needle holders in Sh-L. This method could be utilized for ligation of the coronary artery to provide a myocardial infarction model for experimental use (Figure 4H).

### Applications for epicardial catheter ablation

**Figure 5:**
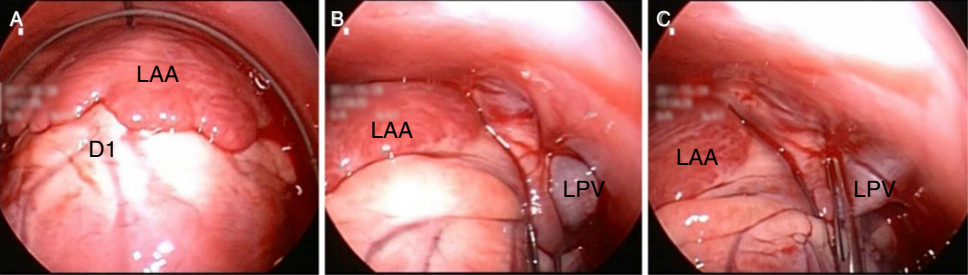
**Applications for epicardial ablation.** The rigid pericardial endoscope-assisted Maze procedure was evaluated. **A**: in the right lateral recumbent position, the left atrial appendage (LAA) was confirmed. **B**: By avoiding LAA by a guide wire, the ostium of the left pulmonary vein (LPV) was identified. **C**: The connection of the left atrium and the ostium of the LPV was securely clamped by forceps without major injury. D1: first diagonal branch; LAA: left atrial appendage; LPV: left pulmonary vein.

Pulmonary veins are known as the site for initiating atrial fibrillation [21]. We evaluated the possibility of isolating the pulmonary vein by rigid pericardial endoscopy. We successfully observed both sides of the pulmonary veins by avoiding the lateral wall of the atrium and the appendage. This was accomplished through the use of forceps and guide wires with the subject in a lateral recumbent position (Figure 5A, B). The pulmonary veins were clamped by forceps without major injuries except for nonsustained atrial fibrillation due to mechanically stretching of the pulmonary veins (Figure 5C). A safe clamp of the pulmonary veins can be a minimally invasive alternative to an open chest Maze procedure.

### Safety of pneumoperitoneum device

Stretching the pericardium with a rigid endoscope could reduce the amount of air in the pericardium that is used for clear visualization. However, filling the pericardial space with air was efficient for this procedure. Similar to laparoscopic surgery, we evaluated the safety of using a continuous pressure-controlled carbon dioxide supply device. The baseline intrapericardial pressure was 2 mmHg, and an additional 3–5 mmHg did not cause any hemodynamic changes. However, an intrapericardial pressure of 6 mmHg induced a sudden systolic pressure drop of 10 mmHg. The fluoroscopy showed a remarkable enlargement of the pericardium, swinging of the heart, and collapse of the left lung. The total amount of supplied air was 700 ml, which suggested that air had leaked into the thoracic cavity and the mediastinum. The pressure drop quickly recovered upon removal of the air. The pressure-controlled air infusion device was not suitable for pericardial endoscopy and required modifications for safe use.

### Safety evaluation

As a promising minimally invasive approach to the pericardial space, the safety of rigid pericardial endoscopy was investigated. With regards to acute complications, arterial blood pressure, and pulse oximetry data were stable during the entire session except for during the use of a continuous air supply device. Other mechanical injuries, such as major bleeding, pneumothorax, and fatal arrhythmia were not identified during these sessions.

## Discussion

### Safer, easier, and clearer: The benefits compared to flexible pericardial endoscopes

The pericardial approach by using rigid pericardial endoscopy was feasible for cell transplantation, pacemaker lead implantation, and epicardial ablation. The efficient use of flexible pericardial endoscopy was previously reported [19]. The use of air to inflate the pericardial space and fluoroscopic guidance of endoscopes along the pericardium enabled us to perform minor surgery in the pericardial space. Compared to flexible pericardial endoscopy, rigid pericardial endoscopy uses smaller devices, more intuitive manipulation techniques, a clearer and wider field of view, a more easily established orientation, easier access to the posterior wall of the heart, and better control of motion artifacts (Table 1). With regards to the approach to the apex, a flexible pericardial endoscope might be easier. Because the flexible pericardial endoscopes were turned around at the roof of the pericardium, we were able to observe the apex in the “inverted U-shape configuration”, as mentioned in the previous paper [19]. The apical view using a rigid pericardial endoscope may lead to an unexpected extraction of the device from the pericardial space, which was successfully avoided by clamping the guide wire.

**Table 1:** Comparisons between rigid and flexible pericardial endoscopes

	**Rigid endoscope**	**Flexible endoscope**
Device size	Smaller	Larger
Manipulation	Intuitive	Fluoroscopy dependent
View	Wider	Position dependent
Orientation	Easier	Fluoroscopy dependent
Lateral and Posterior approach	Easily performed by body position change	Difficult but performed with fluoroscopy
Apical approach	Risk of unintentional extraction	Easily controlled with fluoroscopy
Motional artifact	Fixed by forceps	Reduced by forceps

Compared with flexible pericardial endoscopes, the smaller rigid pericardial endoscopes are more advantageous. These devices are more intuitively manipulated, offer a clearer and wider field of view, are easier to establish orientation, offer easier access to the posterior wall of the heart including the pulmonary veins, and are better at controlling motion artifacts. The use of less air in the pericardial space through stretching the pericardium with rigid endoscopes reduces the risk of complications.

One of the complications caused by flexible pericardial endoscopy was the air tamponade. For easier control, we tried to use the pneumoperitoneum device. However, because the device was maintained by pressure control, the amount of air required was large. An air leak could be lethal, inducing pneumomediastinum and pneumothorax. Therefore, stretching the pericardium with the rigid pericardial endoscopes and retaining the stiff guide wire in the pericardium were more effective in establishing a visual field with a reduced dependence on air.

### Future perspectives

Our study demonstrated that minor surgery for cell transplantation, lead transplantation, and epicardial ablation as assisted by rigid pericardial endoscopy was safe and feasible (Additional file 1). Our methodology of cell transplantation was as safe as open chest surgery. The coronary vessels were avoided macroscopically, and the depth of the needle and the details of the anatomy were confirmed by ultrasonography. This method will soon be performed in clinical practice and will be applicable for gene transfer or cytokine administration. A direct injection of cells might injure the myocardium compared to cell sheet delivery [22]; however, sheet delivery requires open chest surgery. Although the effectiveness of cell sheet delivery for the fat-rich human heart is still unknown, the rigid pericardial endoscope could be applicable for delivering cell sheets less invasively. However, minimally invasive delivery of devices into the pericardial space is difficult, especially for fragile objects such as cell sheets. Further experiments are necessary to explore alternative delivery methods for cell-loaded devices. Once the most effective method of cell transplantation is established, this technology should be applicable for any of these aforementioned applications.

Lead implantation using rigid pericardial endoscopy was possible without any major complications. Because the most appropriate site of lead transplantation in cardiac resynchronizing therapy may not be possible via the coronary sinus, a rigid pericardial endoscopic approach could supply another minimally invasive option. Testing 3 kinds of pacing leads gave us enough information to optimize a prototype for this procedure.

Furthermore, in the field of epicardial ablation, pulmonary veins could be clamped by forceps inserted from the epicardial fossa. The same kind of manipulation was previously performed by thoracoscopy [23]. The less invasive Maze procedure could be performed by a rigid pericardial endoscope. Endoscopic-guided epicardial ablation is advantageous in obtaining gross information on the surface of the heart, especially in cases of arrhythmogenic foci located in the epicaridum near a coronary artery or scar, which requires open chest surgery. Further studies for complicated manipulation, such as penetrating the pericardium, and chronic complications, including pulmonary vein stenosis, should be evaluated.

The recent report showed that transmural atrial gene transfer by painting adenoviral vectors to the epicardial surface was successful [24]. This methodology could be applicable to rigid pericardial endoscopy for safer manipulation. The combination of gene therapy and rigid pericardial endoscopy as a focal transgenic method could cure refractory genetic arrhythmias and ionic channel disorders.

Furthermore, the combination of pericardial endoscopy and thoracoscopy under general anesthesia might enable us to perform more complicated maneuvers; however, that procedure would no longer be minimally invasive. Approaching the epicardial space from the esophagus could be a practical application of natural orifice transluminal endoscopic surgery (NOTES) [25], which could avoid injury to the esophagus during left atrial ablation. Other devices, such as hemostatic devices, suture devices, and epicardial pacemaker lead kits, are now under development. This procedure might be performed after cardiac surgery, and successful adhesiotomy of the pericardium would broaden its indications. We predict that the rigid pericardial endoscopy technique in humans and the broadening of its indications are forthcoming.

### Study limitations

The number of animals was too small to evaluate all of the advantages and disadvantages of this procedure. Because swine and canines were used for this experiment, the anatomical differences between the animal species might result in differences in safety and efficacy. All of the protocols were evaluated for acute complications. Further studies were necessary to evaluate chronic complications and the effectiveness of minor surgery. Furthermore, the risk of infection in cell transplantation and follow-up data of lead function in lead implantation should also be evaluated.

## Conclusions

Rigid pericardial endoscopy is a promising manipulation procedure for minimally invasive cell transplantation, epicardial lead implantation, and epicardial ablation by providing a safe and clear visual field in the pericardial space.

## Competing interests

The authors declare that they have no competing interests.

## Authors’ contributions

TK carried out the experiment and drafted the manuscript. SM participated in the design, coordination and draft of the manuscript. KO, KF, and KT participated in the design of the study and performed the statistical analysis. KS and ST participated in the revision of the manuscript. KF participated in approving the final version of the manuscript. All of the authors read and approved the final manuscript.

## Supplementary Material

Additional file 1 **Video: rigid pericardial endoscopy.** A rigid pericardial endoscope was inserted from the epicardial fossa to the pericardial space. Atrial appendages, coronary sinus and pulmonary veins were clearly identified. As the demonstration of cell transplantation, silicon beads, indocyanide green, ultrasonographic and fluoroscopic contrast medium were injected 20 times. Transient ventricular tachycardia did not deteriorate hemodynamic conditions. The use of an ultrasonography-attached needle was successful in avoiding intraventricular injection. For pacemaker implantation, the left ventricular free wall was clearly visualized in the left recombinant position. The intraventricular pacemaker lead was successfully implanted, and the epicardial sutureless lead and the suture trial were attempted. For epicardial ablation, the left pulmonary vein was identified and clamped by rigid forceps for the minimally invasive Maze procedure. Click here for file
